# High-throughput DNA sequencing of the moose rumen from different geographical locations reveals a core ruminal methanogenic archaeal diversity and a differential ciliate protozoal diversity

**DOI:** 10.1099/mgen.0.000034

**Published:** 2015-10-30

**Authors:** Suzanne L. Ishaq, Monica A. Sundset, John Crouse, André-Denis G. Wright

**Affiliations:** ^1^​ Department of Animal Science, University of Vermont, Burlington, Vermont, USA; ^2^​ Department of Animal and Range Science, Montana State University, Bozeman, Montana, USA; ^3^​ Department of Arctic and Marine Biology, University of Tromsø – The Arctic University of Norway, Tromsø, Norway, USA; ^4^​ Alaska Department of Fish and Game, Soldotna, Alaska, USA; ^5^​ School of Animal and Comparative Biomedical Sciences, University of Arizona, Tucson, Arizona, USA

**Keywords:** methanogens, ciliate protozoa, moose, rumen, high-throughput sequencing

## Abstract

Moose rumen samples from Vermont, Alaska and Norway were investigated for methanogenic archaeal and protozoal density using real-time PCR, and diversity using high-throughput sequencing of the 16S and 18S rRNA genes. Vermont moose showed the highest protozoal and methanogen densities. Alaskan samples had the highest percentages of *Methanobrevibacter smithii*, followed by the Norwegian samples. One Norwegian sample contained 43 % *Methanobrevibacter thaueri*, whilst all other samples contained < 10 %. Vermont samples had large percentages of *Methanobrevibacter ruminantium*, as did two Norwegian samples. *Methanosphaera stadtmanae* represented one-third of sequences in three samples. Samples were heterogeneous based on gender, geographical location and weight class using analysis of molecular variance (AMOVA). Two Alaskan moose contained >70 % *Polyplastron multivesiculatum* and one contained >75 % *Entodinium* spp. Protozoa from Norwegian moose belonged predominantly (>50 %) to the genus *Entodinium*, especially *Entodinium caudatum*. Norwegian moose contained a large proportion of sequences (25–97 %) which could not be classified beyond family. Protozoa from Vermont samples were predominantly *Eudiplodinium rostratum* (>75 %), with up to 7 % *Diploplastron affine*. Four of the eight Vermont samples also contained 5–12 % *Entodinium* spp. Samples were heterogeneous based on AMOVA, principal coordinate analysis and UniFrac. This study gives the first insight into the methanogenic archaeal diversity in the moose rumen. The high percentage of rumen archaeal species associated with high starch diets found in Alaskan moose corresponds well to previous data suggesting that they feed on plants high in starch. Similarly, the higher percentage of species related to forage diets in Vermont moose also relates well to their higher intake of fibre.

## Data Summary

1. 16S rRNA archaeal sequences can be found in the Sequence Read Archive; BioProject PRJNA281249 (http://www.ncbi.nlm.nih.gov/bioproject/281249).
2. 18S rRNA protozoal sequences can be found in the Sequence Read Archive; BioProject PRJNA281109 (http://www.ncbi.nlm.nih.gov/bioproject/281109).

## Impact Statement

For the first time, to the best of our knowledge, the methanogenic archaea in the moose rumen have been identified. Additionally, both methanogens and protozoa diversity have been compared from the rumens of moose across three geographical locations using high-throughput techniques. Rumen ciliate protozoa and methanogenic archaea often form symbiotic relationships for the transport of hydrogen from protozoa to methanogens. Understanding their impact to the moose rumen microbiota is critical to understanding the overall rumen function of wild moose. This information can be applied to further studies on methane production in rumens and potential mitigation strategies, such as altering the methanogen or protozoal diversity to reduce methane output. Surprisingly, there was low methanogen diversity and high protozoal diversity across moose in different locations where they have consumed different diets. Additionally, a large percentage of protozoal sequences could not be identified beyond genus or family, indicating a need for additional work into identifying novel species.

## Introduction

Previous investigations into the micro-organisms in the rumen of the moose have focused on bacteria using cultivation (Dehority, 1986) and high-throughput sequencing techniques (Ishaq & Wright, 2012, 2014a) or on protozoa using light microscopy (Dehority, 1974; Krascheninnikow, 1955; Sládeček, 1946; Westerling, 1969) and high-throughput sequencing (Ishaq & Wright, 2014b). Methanogenic archaea in the rumen of moose have not previously been identified nor have methanogens or protozoa from moose been compared across samples from different geographical locations. Methanogens and protozoa in the rumen are often found in intracellular or extracellular symbiotic associations involving hydrogen transfer from protozoa to methanogens. Previously, protozoa from the genera *Dasytricha*, *Entodinium*, *Polyplastron*, *Epidinium* and *Ophryoscolex* have been shown to interact with methanogens from the orders *Methanobacteriales* and *Methanomicrobiales* (Finlay *et al.*, 1994; Newbold *et al.*, 1995; Sharp *et al.*, 1998; Stumm *et al.*, 1982; Vogels *et al.*, 1980).

For domestic livestock, methanogenesis represents a loss of dietary efficiency as compounds such as acetate or hydrogen are sequestered by methanogens instead of being used by the host for production (i.e. live weight gain, milk production, wool production, etc.). Much research has been performed on methanogenesis and rumen microbial populations between domestic and wild ruminants, as wild ruminants (bison, elk and deer) are estimated to produce up to 0.37 Tg CO_2_ Eq year^− 1^ (Hristov, 2012; McAllister *et al.*, 1996). This is a drastically lower figure than that for domestic livestock, at 141 Tg CO_2_ Eq year^− 1^ (EPA, 2014). Wild ruminants are presumed to produce less methane based on a presumed higher dietary efficiency and lower production demands. As a first step to better understanding methanogenesis in moose, the present study identified the methanogens present in the rumen of moose, as well as the protozoa that are potentially symbiotically associated with them.

The objectives of this research were to identify the methanogens and protozoa present in the rumen of moose from Alaska, Vermont and Norway; to measure the density of methanogens and protozoa in these samples; to compare samples across geographical location, gender and weight class to determine possible trends; and to compare samples with published studies on wild and domesticated ruminants. It was hypothesized that moose may have fewer total methanogens than domestic ruminants due to a fast rate of passage through the gastrointestinal tract (Lechner *et al.*, 2010). In previous studies, age (Godoy-Vitorino *et al.*, 2010; Ishaq & Wright, 2014a; Li *et al.*, 2012) and geographical location (Ishaq & Wright, 2014a; Sundset *et al.*, 2007) have played a role in differentiating core bacterial microbiomes of various hosts, and it was also hypothesized that this would hold true for methanogens in the moose rumen. However, reindeer, which often share a similar diet or geographical location to moose, have been shown to have similar protozoal diversity across geographical locations, indicating that the host species may not have been isolated long enough to develop a unique profile regardless of geographical location of the host (Imai *et al.*, 2004). As moose have not been isolated long, it was hypothesized that this would hold true for moose as well.

## Methods

A total of 17 rumen samples were collected from wild moose in Vermont, USA (*n* = 8) (October 2010), Troms County, Norway (*n* = 6) (September–October 2011) and captive wild moose in Soldotna, Alaska, USA (*n* = 3) (August 2012). Sample collection and DNA extraction were described previously (Ishaq & Wright, 2014a). Briefly, fresh-frozen whole rumen samples from Vermont and ethanol-fixed samples from Norway were collected during field dressing of carcasses by hunters, and ethanol-fixed Alaskan samples were collected via oesophageal tubing (Institutional Animal Care and Use Committee protocol 11-021, University of Vermont; Animal Care and Use Committee protocol 2011-026, Department Fish and Game, Alaska). Metadata for each sample collected, including gender, weight, approximate age and coordinates of sample collection, have also been published elsewhere (Ishaq & Wright, 2014a). Pooled samples from Alaska (*n* = 3) were previously sequenced and described (Ishaq & Wright, 2014b). Samples were identified by location (Alaska, AK; Norway, NO; Vermont, VT), host (m, moose), individual moose (1–8) and sample material (r, rumen), consistent with previous publications (Ishaq & Wright, 2012, 2014a, b).

PCR was performed on a C1000 ThermalCycler (Bio-Rad) using a Phusion kit (ThermoScientific) to amplify rDNA. For methanogenic archaea, the V1–V3 region of the archaeal 16S rRNA gene was amplified using primers 86F (5′-GCTCAGTAACACGTGG-3′) (Wright *et al.*, 2004) and 471R (5′-GWRTTACCGCGGCKGCTG-3′) (Cersosimo *et al.*, 2015). The protocol was as follows: initial denaturing at 98 °C for 10 min, then 35 cycles of 98 °C for 30 s, 58 °C for 30 s and 72 °C for 30 s, then a final elongation step of 72 °C for 6 min. For ciliate protozoa, the V3–V4 and signature regions 1–2 of the 18S rRNA gene were amplified using primers P-SSU-316F (5′-GCTTTCGWTGGTAGTGTATT-3′) (Sylvester *et al.*, 2004) and GIC758R (5′-CAACTGTCTCTATKAAYCG-3′) (Ishaq & Wright, 2014b) as described previously (Ishaq & Wright, 2014b). PCR amplicons were verified on a 1 % agarose gel (100 V, 60 min), and DNA bands were excised and purified as described previously (Ishaq & Wright, 2014a). Amplicons were sent to MR DNA Laboratories for Illumina MiSeq version 3 (methanogens) or Roche 454 pyrosequencing with Titanium (protozoa).

### Sequence analysis

All sequences were analysed using mothur version 1.31 (Schloss *et al.*, 2009) and are available under the NCBI Sequence Read Archive under Bioproject IDs PRJNA281249 for methanogens and PRJNA281109 for protozoa. For methanogens, sequence analysis was as described previously (Ishaq & Wright, 2014a), with the following modifications. Sequences were trimmed to a uniform length of 436 alignment characters (minimum 350 bases) and candidate sequences were aligned against the Ribosomal Database Project reference alignment integrated into mothur with the bacterial sequences removed. Sequences were classified using the *k*-nearest-neighbour method against the full Ribosomal Database Project alignment, which had been modified to include species-level taxonomy. A 2 % genetic distance cut-off was used to designate species. For protozoa, sequence analysis was as previously described for primer set 1 (PSSU316F and GIC758R), using a 4 % genetic distance cut-off to designate species (Ishaq & Wright, 2014b). Sequences were subsampled evenly for each sample. The operational taxonomic unit (OTU) estimators CHAO (Chao & Shen, 2003) and ACE (http://chao.stat.nthu.edu.tw), Good's Coverage (Good, 1953), and the Shannon–Weaver Diversity Index (Shannon & Weaver, 1949) were calculated. An analysis of molecular variance (AMOVA) and UniFrac (Hamady *et al.*, 2010) were used to compare the heterogeneity of samples. UniFrac measures overall phylogenetic tree distance between samples and will create a dendrogram which clusters samples. Principal coordinate analysis (PCoA) calculates the distance matrix for each pair of samples and then turns these distances into points in a space with a number of dimensions one less than the number of samples.

### Real-time (RT)-PCR

RT-PCR was used to calculate archaeal and protozoal densities in whole samples. DNA was amplified using a CFX96 Real-Time System (Bio-Rad) and a C1000 ThermalCycler (Bio-Rad). Data were analysed using CFX Manager Software version 1.6 (Bio-Rad). An iQ SYBR Green Supermix kit (Bio-Rad) was used: 12.5 μl mix, 2.5 μl each primer (40 mM), 6.5 μl H_2_O and 1 μl initial DNA extract (Ishaq & Wright, 2014a) diluted to ∼10 ng μl^− 1^. For methanogens, the primers targeted the methyl coenzyme M reductase A gene (*mrcA*), mcrA-F 5′-GGTGGTGTMGGATTCACACAGTAYGC-3′ and mcrA-R 5′-TTCATTGCRTAGTTWGGRTAGTT-3′, following the protocol of Denman *et al.* (2007). The internal standards for methanogens were a mix of *Methanobrevibacter smithii*, *Methanobrevibacter gottschalkii*, *Methanobrevibacter ruminantium* and *Methanobrevibacter millerae* (*R*^2^ = 0.998).

For protozoa, the primers PSSU316F and PSSU539R (5′-ACTTGCCCTCYAATCGTWCT-3′) (Sylvester *et al.*, 2004) targeted the 18S rRNA gene, following the protocol by Sylvester *et al.* (2004), and internal standards for protozoa were created in the laboratory using fresh dairy cattle rumen contents which were filtered through one layer of cheesecloth to remove large particles, and then the protozoa were allowed to separate for 2 h at 39 °C. Once a protozoal pellet was visible, 50 ml was drawn from the bottom of the funnel and 1 vol. 100 % ethanol was added to fix the cells and DNA. The mix was centrifuged for 5 min at 2000 ***g***, and the pellet was washed with TE buffer (1 M Tris/HCl, 0.5 M EDTA, pH 8.0) and then centrifuged again. Cells were counted microscopically using a Thoma Slide following the protocol by Dehority (1974) (*R*^2^ = 0.998). Both protocols were followed by a melt curve, with a temperature increase of 0.5 °C every 10 s from 65 °C up to 95 °C to check for contamination.

## Results

### Methanogens

**Fig. 1.**
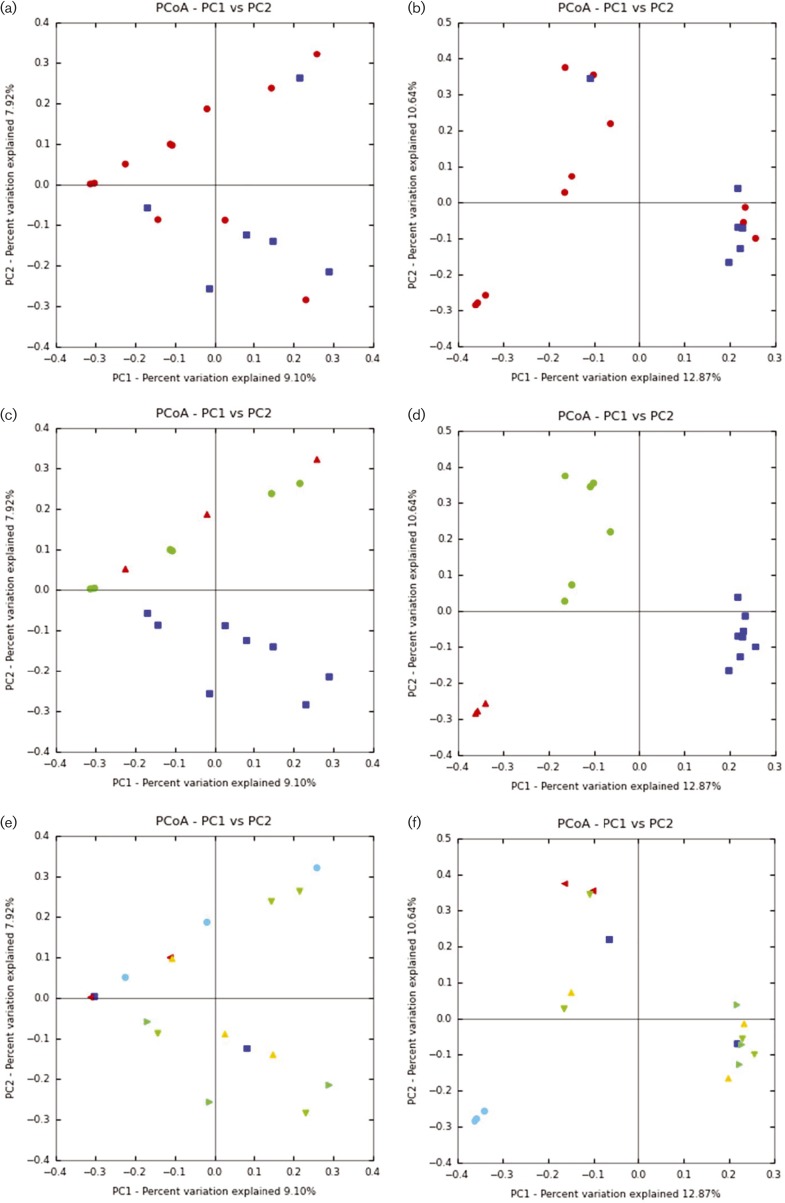
PCoA for moose methanogens (A, C, E) and protozoa (B, D, F). PCoA is coloured by (A, B) gender: female, red; male, blue; (C, D) location: Alaska, red; Norway, green; Vermont, blue; and (E, F) weight class: 1–100 kg, red triangle; 101–200 kg, yellow triangle; 201–300 kg, green down-facing triangle; 301–400 kg, green right-facing triangle, >400 kg (live weight), light blue circle; not available, blue square.

A total of 141 368 sequences, of which 47 370 were unique, passed quality assurance steps. For each sample, between 22 and 330 OTUs were assigned using a 2 % genetic distance cut-off (Wright *et al.*, 2009), giving a total of 1942 non-redundant OTUs. CHAO, ACE, Good's Coverage and Shannon–Weaver Diversity Index for each sample are provided in Table 1. The Vermont samples showed the highest Shannon–Weaver Diversity Index, CHAO and ACE, whilst the Norwegian samples showed the highest Good's Coverage. The Alaskan samples showed the highest observed OTUs. Although there were few shared OTUs amongst samples, these shared OTUs represented a large number of shared sequences (Table 2). Comparing all 17 samples across different factors using AMOVA, groups were heterogeneous based on gender (*P* < 0.001), geographical location (*P* < 0.001) and weight class (*P* < 0.001). Samples were significantly different from each other by AMOVA (*P* < 0.001), except for VTM1R and VTM2R (*P* = 0.052). In contrast, samples did not cluster significantly based on gender or weight class using PCoA (Fig. 1A, E), although Vermont clustered separately from Norway and Alaska (Fig. 1C). When comparing samples using UniFrac, all samples again did not cluster significantly using either weighted (mean 0.17, range 0.05–0.28, *P* < 0.001) or unweighted (mean 0.91, range 0.84–0.96, *P* = 0.20) parameters. However, 16 out of 136 pairwise sample comparisons were significantly different (*P* < 0.001). Whilst fresh-frozen and ethanol-fixed did cluster somewhat separately with methanogen samples on PCoA (PC1 versus PC2), this was not seen with PC3 versus PC2 or with PC3 versus PC1 (data not shown).

**Table 1.** Statistical measures per sample for methanogens and protozoa in Alaska, Norway and Vermont Samples were subsampled using the smallest group for methanogens and protozoa. Species-level cut-off was 2 % for methanogens and 4 % for protozoa.

Sample	Total sequences	Total OTUs	Subsampled sequences				
			OTUs	CHAO	ACE	Good's Coverage	Shannon–Weaver Diversity Index
**Methanogens**							
AKM1R	4366	152	18	161	14	0.93	0.47
AKM2R	537	23	20	200	0	0.92	0.52
AKM3R	53 648	292	6	17	10	0.98	0.15
*Mean*	*19* *517*	*156*	*15*	*126*	*8*	*0.94*	*0.38*
NOM1R	506	22	22	232	0	0.91	0.56
NOM2R	1830	79	19	163	26	0.93	0.48
NOM3R	19 990	70	6	16	5	0.98	0.14
NOM4R	1355	33	15	115	0	0.94	0.39
NOM5R	17 130	293	12	56	39	0.96	0.30
NOM6R	7106	70	9	32	41	0.97	0.24
*Mean*	*7986*	*95*	*14*	*102*	*19*	*0.95*	*0.35*
VTM1R	2351	102	26	262	328	0.90	0.70
VTM2R	1803	63	23	213	105	0.91	0.59
VTM3R	12 855	99	11	46	111	0.96	0.31
VTM4R	4477	149	22	219	47	0.91	0.56
VTM5R	1180	82	38	470	675	0.85	1.02
VTM6R	3142	155	29	325	275	0.89	0.77
VTM7R	790	43	28	389	0	0.89	0.73
VTM8R	8302	330	31	359	538	0.88	0.81
*Mean*	*4363*	*128*	*26*	*285*	*260*	*0.90*	*0.69*
**Protozoa**							
AKM1R	81 387	31	1	1	0	1.00	0.01
AKM2R	57 698	31	1	1	0	1.00	0.01
AKM3R	16 200	12	1	1	0	1.00	0.01
*Mean*	*51* *762*	*25*	*1*	*1*	*0*	*1.00*	*0.01*
NOM1R	24 605	1	1	1	0	1.00	0.00
NOM2R	15 468	4	2	2	0	0.99	0.05
NOM3R	20 351	9	3	4	0	0.97	0.14
NOM4R	5354	1	1	1	0	1.00	0.00
NOM5R	35 189	7	2	2	0	0.99	0.06
NOM6R	30 145	7	2	2	0	0.99	0.08
*Mean*	*21* *852*	*5*	*2*	*2*	*0*	*0.99*	*0.06*
VTM1R	24 635	2	2	2	0	0.99	0.08
VTM2R	27 901	6	2	2	0	0.99	0.06
VTM3R	41 131	8	4	7	2	0.96	0.25
VTM4R	27 612	3	1	1	0	0.99	0.03
VTM5R	30 065	5	3	4	0	0.97	0.15
VTM6R	8334	3	3	3	0	0.98	0.12
VTM7R	27 742	2	2	2	0	0.99	0.04
VTM8R	25 335	1	1	1	0	1.00	0.00
*Mean*	*26* *594*	*4*	*2*	*3*	*0*	*0.98*	*0.09*

**Table 2.** The number of shared OTUs and unique sequences across different samples in Alaska, Norway and Vermont Cut-off values of 2 % for methanogens and 4 % for protozoa were used to generate OTUs.

Samples	Methanogens		Protozoa	
compared	OTUs	Unique sequences	OTUs	Unique sequences
All samples (*n* = 17)	1	44 967	1	48 850
Alaska samples (*n* = 3)	2	19 888	2	45 572
Norway samples (*n* = 6)	2	16 227	2	1771
Vermont samples (*n* = 8)	2	11 255	2	1635
All females (*n* = 11)	2	31 300	2	47 400
All males (*n* = 6)	2	16 070	2	1578
0–100 kg (NOM1R, NOM6R)	2	2684	2	519
101–200 kg (NOM2R, VTM1R, VTM3R)	4	4804	2	528
201–300 kg (NOM3R, NOM5R, VTM2R, VTM6R)	2	14 007	2	1384
301–400 kg (VTM5R, VTM7R, VTM8R)	2	3729	2	541

**Fig. 2.**
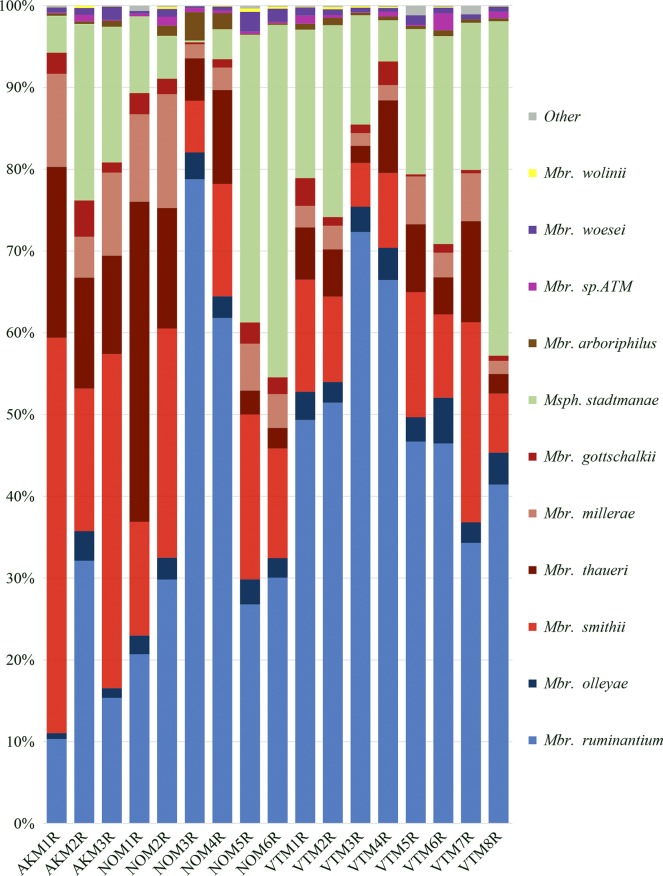
Diversity of moose rumen methanogens. Members of the RO clade are coloured in blues; members of the SGMT clade are coloured in reds. *Mbr.*, *Methanobrevibacter*.

Vermont samples contained the highest mean density of methanogens at 1.3e+10 cells ml^–1^, followed by Alaskan samples and Norwegian samples (5.19e+09 and 3.58e+09 cells ml^–1^, respectively) (Table 3). Whilst there was a positive correlation between individual methanogen and protozoal density in moose, it was not significant (*R*^2^ = 0.38) (data not shown). Two of three Alaskan moose, as well as two of six Norwegian moose had a larger proportion of methanogens belonging to the SGMT clade (*Methanobrevibacter* *smithii*, *Methanobrevibacter* *gottschalkii*, *Methanobrevibacter* *millerae*, and *Methanobrevibacter* *thauri*). All eight Vermont moose, one Alaskan moose and four Norwegian moose had greater proportions of members of the RO clade (*Methanobrevibacter* *ruminantium* and *Methanobrevibacter* *olleyae*) (Fig. 2). There was also no trend seen between moose age and methanogen density (*R*^2^ = 0.015, data not shown).

**Table 3.** RT-PCR results for methanogenic archaea and ciliate protozoa in Alaska, Norway and Vermont

Sample	Corrected cells (ml rumen digesta)^− 1^	
	Archaea	Protozoa
AKM1R	3.33e+09	3.60e+06
AKM2R	1.91e+09	4.72e+05
AKM3R	1.03e+10	7.43e+06
*Mean (se)*	*5.19e+09 (4.51e+09)*	*3.83e+06 (3.48e+06)*
NOM1R	8.66e+07	5.92e+03
NOM2R	1.54e+08	1.10e+04
NOM3R	1.95e+08	5.46e+04
NOM4R	1.38e+08	7.26e+03
NOM5R	2.17e+10	1.67e+05
NOM6R	8.25e+08	6.45e+04
*Mean (se)*	*3.58e+09 (8.76e+09)*	*5.17e+04 (6.20e+04)*
VTM1R	3.87e+09	2.14e+06
VTM2R	1.88e+10	3.00e+06
VTM3R	4.26e+10	9.02e+06
VTM4R	1.98e+10	5.70e+06
VTM5R	7.68e+09	6.53e+06
VTM6R	8.93e+08	5.08e+05
VTM7R	4.54e+09	5.50e+06
VTM8R	6.02e+09	5.18e+06
*Mean (se)*	*1.3e+10 (1.38e+10)*	*4.70e+06 (2.70e+06)*
*Mean all (se)*	*7.36e+09 (4.95e+09)*	*2.86e+06 (2.47e+06)*

Alaskan samples had the highest percentages of *Methanobrevibacter smithii* (16–36 %), followed by the Norwegian samples (10–24 %) (Fig. 2). The Norwegian sample NOM1R contained the highest percentage of *Methanobrevibacter thaueri* (43 % of total sequences), whilst all other samples contained < 10 %. Vermont samples had large percentages of *Methanobrevibacter ruminantium* (27–51 % of total sequences), as did the Norwegian samples NOM3R and NOM4R (40 and 41 %, respectively) (Fig. 2). *Methanosphaera stadtmanae* was highest in NOM5R (36 %), VTM8R (35 %) and NOM6R (34 %) (Fig. 2). Less than 36 sequences total were found of each of the following: *Methanocella*, *Methanospirillum*, *Methanolobus*, *Methanosarcina*, *Picrophilus*, *Methanobacterium*, *Methanobrevibacter curvatus*, *Methanobrevibacter cuticularis* or unclassified at the genus level (‘Other’; Fig. 2).

### Protozoa

A total of 499 152 sequences, of which 72 091 were unique, passed quality assurance steps. For each sample, between 1 and 31 OTUs were estimated using a 4 % genetic distance cut-off (Ishaq & Wright, 2014b), giving a total of 110 non-redundant OTUs. CHAO, ACE, Good's Coverage and Shannon–Weaver Diversity Index for each sample are provided in Table 1. Both Norwegian and Vermont samples had extremely high coverage (>0.97 %), yet low Shannon–Weaver Diversity Index, CHAO and ACE values. Although there were few shared OTUs amongst samples, these shared OTUs represented a large number of shared sequences (Table 2). When comparing samples using UniFrac, samples clustered significantly using weighted (mean 0.71, range 0.09–0.99, *P* < 0.001) and unweighted (mean 0.93, range 0.75–0.99, *P* < 0.001) parameters. When comparing the Norway and Vermont samples across different factors using AMOVA, groups were heterogeneous based on gender (*P* < 0.001), geographical location (*P* < 0.001) and weight class (*P* < 0.001). Samples were significantly different from each other (*P* < 0.05) using AMOVA, with the exception of the following within-group pairwise comparisons: VTm4Rprot–VTm8Rprot, VTm3Rprot–VTm7Rprot, VTm2Rprot–VTm4Rprot, VTm1Rprot–VTm7Rprot, VTm1Rprot–VTm6Rprot and VTm1Rprot–VTm3Rprot (*P*>0.05). This was also confirmed using PCoA for gender, location, and weight class (Fig. 1B, D, F). No clustering bias was seen with respect to storage technique of samples.

Vermont samples contained the highest mean density of protozoa at 4.70e+06 cells ml^–1^, followed by Alaskan samples and Norwegian samples (3.83e+06 and 5.17e+04 cells ml^–1^, respectively) (Table 3). Whilst there was a positive correlation between individual methanogen and protozoal density in moose, it was not significant (*R*^2^ = 0.38) (Data not shown). There was also no trend seen between moose age and protozoal density (*R*^2^ = 0.107, data not shown).

**Fig. 3.**
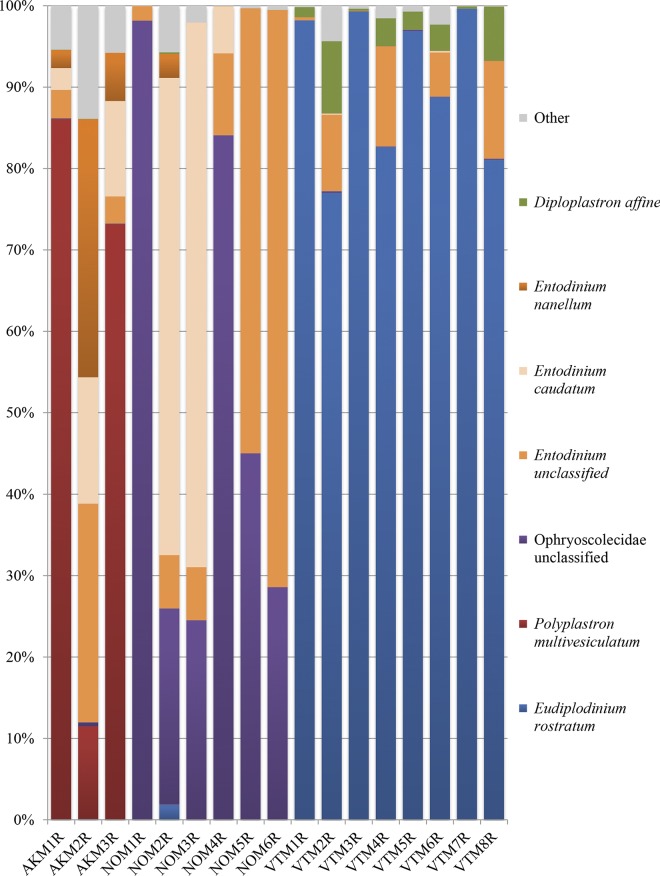
Diversity of the moose rumen protozoa.

Protozoa were identified using a previously described reference alignment and taxonomy of valid protozoal sequences (Ishaq & Wright, 2014b) (Fig. 3). Two Alaskan moose contained >70 % *Polyplastron multivesiculatum* and one contained >75 % *Entodinium* spp. Protozoa from Norwegian moose belonged predominantly (>50 % of total sequences) to the genus *Entodinium*, especially *Entodinium caudatum* (Fig. 3). A large proportion of sequences in Norwegian moose (25–97 % of total sequences) could not be classified beyond the family Ophryoscolecidae (Fig. 3). Protozoa from Vermont samples were predominantly composed of *Eudiplodinium rostratum* (>75 % of total sequences). Vermont samples also contained up to 7 % *Diploplastron affine* (Fig. 3). Many other species were identified in moose, with < 1 % each of the following identified: *Anoplodinium denticulatum*, *Dasytricha* spp., *Diplodinium dentatum*, *Enoploplastron triloricatum*, *Entodinium bursa*, *Entodinium dubardi*, *Entodinium furca dilobum*, *Entodinium furca monolobum*, *Entodinium longinucleatum*, *Entodinium simplex*, *Epidinium caudatum*, *Epidinium ecaudatum caudatum*, *Epidinium* spp., *Eremoplastron dilobum*, *Eremoplastron rostratum*, *Eudiplodinium maggii*, *Isotricha intestinalis*, *Isotricha prostoma*, *Metadinium medium*, *Metadinium minorum*, *Ophryoscolex purkynjei*, *Ophryoscolex* spp., *Ostracodinium clipeolum*, *Ostracodinium dentatum*, *Ostracodinium gracile* and *Ostracodinium* spp.

## Discussion

### Geographical location

The present study represents the first insight into the methanogenic archaeal diversity in the rumen of the moose. Although distinct in terms of proportion of methanogenic taxa present in each of the three moose populations, the samples were not statistically different between geographical populations. This suggests that moose have a core methanogen microbiome, as has been suggested for protozoa in other host species (Imai *et al.*, 2004). It is possible that, whilst diet is a significant factor in determining the micro-organisms present in the rumen, there are other factors, such as body/rumen temperature or rumen pH, which are selecting for similar methanogen species in moose from different geographical locations on different diets. There was also no trend seen between moose age and methanogen density in the present study, despite clear trends between age and density in previous studies (Saengkerdsub *et al.*, 2007; Skillman *et al.*, 2004). This may be due to a relatively small sample size or trends may be indistinct once the moose rumen reaches developmental maturity before its first year.

Given the markedly different protozoal populations found in Alaska, Vermont and Norway, as well as the AMOVA analysis confirming statistically different groups, it may be concluded that moose do not have a typical protozoal diversity as do reindeer from various locations (Imai *et al.*, 2004). It is estimated that moose reached North America across the Bering Strait from Asia some 14 000–11 000 years ago, but it was not until relatively recently that moose dispersed to peripheral (i.e. coastal) regions and began to diversify genetically (Hundertmark *et al.*, 2003). Despite the relatively recent diversification of moose subspecies in North America, moose have been geographically isolated long enough to form distinct rumen protozoal populations.

Two of three Alaskan moose, as well as two of six Norwegian moose, had a larger proportion of methanogens belonging to the SGMT clade (*Methanobrevibacter* *smithii*, *Methanobrevibacter* *gottschalkii*, *Methanobrevibacter* *millerae* and *Methanobrevibacter* *thauri*). All eight Vermont moose, one Alaskan moose and four Norwegian moose had greater proportions of members of the RO clade (*Methanobrevibacter* *ruminantium* and *Methanobrevibacter* *olleyae*). Previously, the SGMT clade was shown to be prevalent in alpaca (St-Pierre & Wright, 2012), sheep (Wright *et al.*, 2008) and Svalbard reindeer (*Rangifer tarandus platyrhynchus*) (Sundset *et al.*, 2009a), as well as Norwegian reindeer (*Rangifer tarandus tarandus*) (Sundset *et al.*, 2009b).

As with moose rumen bacteria in a previous study (Ishaq & Wright, 2014a), Alaskan moose shared a large number of methanogenic sequences, followed by the Norwegian samples, and females shared more archaeal and protozoal sequences than males. Unlike previously (Ishaq & Wright, 2014a), the 202–300 kg weight class shared the greatest number of archaeal and protozoal sequences of all the weight classes. Whilst the total methanogen OTUs were higher than usually reported, our numbers were not outside the range of those previously reported for other ruminants (7168 OTUs, with a range of 788–2758 OTUs; Piao Hailan *et al.*, 2014). The present study retained singletons and doubletons to prevent the removal of rare taxa.

### Diet

*Methanosphaera stadtmanae* has previously been associated with diets including fruit, as they require methanol (a byproduct of pectin fermentation), and has been previously seen in omnivores (Dridi *et al.*, 2009; Facey *et al.*, 2012) and the rumen of various hosts (Cersosimo *et al.*, 2015; Cunha *et al.*, 2011; Snelling *et al.*, 2014). Nordic blueberries have been found in the diet of Norwegian moose (Shipley *et al.*, 1998; Wam & Hjeljord, 2010) and often bear fruit year-round. Nordic blueberries contain an average of 0.7 g fat and it is possible that an increase in dietary fat from berries reduced methanogen density (Dohme *et al.*, 2001) in Norwegian moose in the present study.

*Methanobrevibacter smithii*, unlike many other methanogens, has been shown to grow at less than neutral pH (Rea *et al.*, 2007), is often associated with high-calorie diets in ruminants (Carberry *et al.*, 2014; Zhou *et al.*, 2010), has been associated with high-efficiency animals (Zhou *et al.*, 2010), has been shown to influence weight gain in rats (Mathur *et al.*, 2013) and has been shown to improve polysaccharide fermentation by bacteria (Joblin *et al.*, 1990; Samuel & Gordon, 2006). Conversely, *Methanobrevibacter ruminantium* has been associated with a high-forage diet in ruminants (Zhou *et al.*, 2010). Previously, Alaskan moose were speculated to be on a high starch/energy diet and showed a much higher proportion of *Bacteroidetes*, especially *Prevotella* spp. (Ishaq & Wright, 2014a), which are associated with protein and starch digestion in the gastrointestinal tract. *Methanobrevibacter smithii* improves polysaccharide digestion by bacteria (Joblin *et al.*, 1990; Samuel & Gordon, 2006).

Previously, Vermont moose were presumed to be on a high forage/low energy diet (Ishaq & Wright, 2012, 2014a), which may account for the relatively high proportions of *Methanobrevibacter ruminantium* in the present study. Whilst *Methanobrevibacter ruminantium* does not use acetate for methanogenesis, it can use formate, which is created during acetogenesis. An increase in plant cell wall digestion increases the amount of acetate produced in the rumen, which can increase methanogenesis by providing methyl groups (Johnson & Johnson, 1995). Roughage diets in livestock have been shown to increase methane emissions (Liu *et al.*, 2012), even when the roughage diets are not associated with altered methanogen densities (Liu *et al.*, 2012; Zhou *et al.*, 2010).

Previously, domestic steers fed a roughage diet had a mean density of 1.34e+09 cells ml^–1^ for methanogens, which were predominantly *Methanobrevibacter* spp. (Denman *et al.*, 2007), and which was comparable to the present study. Holstein dairy cattle on a high-forage diet had a mean density of 6.04e+05 cells ml^–1^ for protozoa (Sylvester *et al.*, 2004), which is similar to densities in moose. It was also shown that densities decreased on a low-forage diet and the dominant genus was *Entodinium* spp. (Sylvester *et al.*, 2004).

Factors such as diet (Dehority & Odenyo, 2003; Morgavi *et al.*, 2012; Sundset *et al.*, 2009a) and weaning strategy (Naga *et al.*, 1969) have an effect on numbers and type of protozoa. Previously, total protozoal counts were shown to be elevated in concentrate-selector herbivores (Dehority & Odenyo, 2003), whilst *Entodinium* populations were decreased in animals fed a higher concentrate diet over those fed a roughage diet (Dehority & Odenyo, 2003). *Entodinium* spp. are a major source of starch digestion in the rumen, as well as bacterial digestion (Williams & Coleman, 1992). *Polyplastron multivesiculatum* produces xylanase and other carbohydrate-degrading enzymes (Béra-Maillet *et al.*, 2005), which allows it to break down hemicellulose in plant cell walls and contribute to fibre digestion. *Eudiplodinium* spp. also preferentially ingest structural carbohydrates (Hungate, 1942; Michałowski *et al.*, 1991).

### Microbial interactions

*Methanobrevibacter ruminantium*, found in Vermont moose, has previously been associated with higher densities of *Polyplastron*, *Eudiplodinium* and *Entodinium* (Ohene-Adjei *et al.*, 2007; Sharp *et al.*, 1998; Vogels *et al.*, 1980). Vermont samples were dominated by *Polyplastron multivesiculatum* and *Eudiplodinium maggii*, whilst Norwegian samples were dominated by *Entodinium* spp. Previously, using light microscopy, moose were shown to have primarily *Entodinium* spp., including *Entodinium dubardi* and *Entodinium longinucleatum* in Alaska (Dehority, 1974), *Entodinium dubardi* and other *Entodinium* spp. from Slovakia, and *Entodinium dubardi* and *Epidinium caudatum* in Finish Lapland (Westerling, 1969). More recently, using high-throughput sequencing, moose in Alaska were shown to have a high percentage of *Polyplastron multivesiculatum*, and well as a variety of *Entodinium* and other species (Ishaq & Wright, 2014b), which was also shown in the present study. The Norwegian samples had a high percentage of *Entodinium caudatum*, *Entodinium furca dilobum*, and other *Entodinium* species, giving them a similar profile to moose samples from Alaska (Dehority, 1974), Finland (Westerling, 1969) and Slovakia (Sládeček, 1946) using light microscopy. The Norwegian samples also contained a large proportion of sequences which could not be identified beyond the family level, indicating that these moose host novel ciliate species or that no 18S rRNA sequences exist for previously identified species.

It has been shown that protozoal density affects methanogen density (Morgavi *et al.*, 2012; Newbold *et al.*, 1995), as the two microbial communities are often symbiotically associated with one another. In the present study, there was a trend towards positively correlated methanogen and protozoal density in individual moose, but it was not significant (*R*^2^ = 0.38). *Polyplastron*, *Eudiplodinium maggii* and *Entodinium caudatum* have been shown to have >40 % association with methanogens (Vogels *et al.*, 1980). More specifically, *Polyplastron* was recently shown to associate with *Methanosphaera stadtmanae* and *Methanobrevibacter ruminantium* (Ohene-Adjei *et al.*, 2007).

Methanogen and protozoal densities in reindeer from Norway (Sundset *et al.*, 2009b) averaged very closely to densities found in Norwegian moose, which were lower than in Alaskan and Vermont moose. In addition to the possibility that dietary fat was reducing methanogens, another possible reason for the low methanogen density in Norwegian moose is the presence of bacterial competitors (Wright & Klieve, 2011), such as acetate-utilizing, hydrogen-utilizing or sulphate-reducing bacteria, which sequester free CO_2_ and H_2_ in the rumen. Very few sequences of *Acetitomaculum* or *Eubacterium* (acetate-utilizing) were identified, but they were found in Norwegian samples previously (Ishaq & Wright, 2014a). Sulphate-reducing bacteria, many of which belong to the *Clostridium* class of the phylum *Firmicutes*, were previously found in Norwegian reindeer (Sundset *et al.*, 2007), but were not found in abundance in Norwegian moose. Likewise, only a few *Desulfovibrio* spp. were found in Norwegian moose, although the phylum *Proteobacteria* was in largest abundance in Norwegian moose, so perhaps more sulphate-reducing bacteria exist which would not be classified down to family (Ishaq & Wright, 2014a).

However, acetate-producing (acetogens) or hydrogen-producing bacteria could have potentially contributed to a higher methanogen density in Alaskan or Vermont samples. Acetogens, such as the phylum *Actinobacteria*, were found in low quantities across the board, with the exception of NOM4R (6 %) (Ishaq & Wright, 2014a). However, specific acetogens species (members of *Sporomusa*, *Moorella*, *Clostridium*, *Acetobacterium* and *Thermoanaerobacter*) were not identified, and their respective families were not found in large abundance in any sample. Of several known hydrogen-producing genera, *Selenomonas* and *Streptococcus* were found in low numbers, and *Bacteroides* and *Succinomonas* were not found. However, the order *Bacteroidales* was previously found in abundance in Alaskan samples (36–83 % of sequences), Norwegian samples (0.7–54 %) and Vermont samples (7–30 %) (Ishaq & Wright, 2014a).

If Alaskan moose were indeed consuming a diet relatively high in starch, the resulting reduction in pH would not be detrimental to *Methanobrevibacter smithii* density, which would in turn support bacterial polysaccharide fermentation (Joblin *et al.*, 1990; Samuel & Gordon, 2006). Concentrate diets in livestock have been shown to reduce the total number of methanogens by decreasing the pH and increasing the food passage rate; however, even a forage diet higher in starch would not contain enough readily fermentable starches to produce the same effect in these moose.

## References

[mgen000034-Bera-Maillet1] Béra-Maillet C. Devillard E. Cezette M. Jouany J.-P. Forano E. (2005). Xylanases and carboxymethylcellulases of the rumen protozoa *Polyplastron multivesiculatum*, *Eudiplodinium maggii* and *Entodinium* sp FEMS Microbiol Lett 244 149–156 10.1016/j.femsle.2005.01.035 . 15727834

[mgen000034-Carberry1] Carberry C. A. Waters S. M. Kenny D. A. Creevey C. J. (2014). Rumen methanogenic genotypes differ in abundance according to host residual feed intake phenotype and diet type Appl Environ Microbiol 80 586–594 10.1128/AEM.03131-13 . 24212580 PMC3911112

[mgen000034-Cersosimo1] Cersosimo L. M. Lachance H. St-Pierre B. van Hoven W. Wright A.-D. G. (2015). Examination of the rumen bacteria and methanogenic archaea of wild impalas (*Aepyceros melampus melampus*) from Pongola, South Africa Microb Ecol 69 577–585. 25351144 10.1007/s00248-014-0521-3

[mgen000034-Chao1] Chao A. Shen T.-J. (2003). Nonparametric estimation of Shannon's index of diversity when there are unseen species in sample Environ Ecol Stat 10 429–443 10.1023/A:1026096204727.

[mgen000034-Cunha1] Cunha I. S. Barreto C. C. Costa O. Y. A. Bomfim M. A. Castro A. P. Kruger R. H. Quirino B. F. (2011). Bacteria and Archaea community structure in the rumen microbiome of goats (*Capra hircus*) from the semiarid region of Brazil Anaerobe 17 118–124 10.1016/j.anaerobe.2011.04.018 . 21575735

[mgen000034-Dehority1] Dehority B. A. (1974). Rumen ciliate fauna of Alaskan moose (*Alces americana*), musk-ox (*Ovibos moschatus*) and Dall moutain sheep (*Ovis dalli*) J Protozool 21 26–32 10.1111/j.1550-7408.1974.tb03612.x . 4206407

[mgen000034-Dehority12] Dehority B. A. (1986). Microbes in the foregut of arctic ruminants. In Control of Digestion and Metabolism in Ruminants: Proceedings of the Sixth International Symposium on Ruminant Physiology, pp. 307–325. Edited by Milligan L. P. Grovum W. L. Dobson A. Englewood Cliffs, NJ Prentice-Hall.

[mgen000034-Dehority123] Dehority B. A. Odenyo A. A. (2003). Influence of diet on the rumen protozoal fauna of indigenous African wild ruminants J Eukaryot Microbiol 50 220–223 10.1111/j.1550-7408.2003.tb00121.x . 12836880

[mgen000034-Denman1] Denman S. E. Tomkins N. W. McSweeney C. S. (2007). Quantitation and diversity analysis of ruminal methanogenic populations in response to the antimethanogenic compound bromochloromethane FEMS Microbiol Ecol 62 313–322 10.1111/j.1574-6941.2007.00394.x . 17949432

[mgen000034-Dohme1] Dohme F. Machmüller A. Wasserfallen A. Kreuzer M. (2001). Ruminal methanogenesis as influenced by individual fatty acids supplemented to complete ruminant diets Lett Appl Microbiol 32 47–51 10.1046/j.1472-765x.2001.00863.x . 11169041

[mgen000034-Dridi1] Dridi B. Henry M. El Khéchine A. Raoult D. Drancourt M. (2009). High prevalence of *Methanobrevibacter smithii**Methanosphaera stadtmanae* detected in the human gut using an improved DNA detection protocol PLoS One 4 e7063 10.1371/journal.pone.0007063 . 19759898 PMC2738942

[mgen000034-EPA1] EPA (2014). Inventory of US Greenhouse Gas Emissions and Sinks: 1990–2012 Washington, DC Environmental Protection Agency.

[mgen000034-Facey1] Facey H. V. Northwood K. S. Wright A.-D. G. (2012). Molecular diversity of methanogens in fecal samples from captive Sumatran orangutans (*Pongo abelii*) Am J Primatol 74 408–413 10.1002/ajp.21992 . 22511523

[mgen000034-Finlay1] Finlay B. J. Esteban G. Clarke K. J. Williams A. G. Embley T. M. Hirt R. P. (1994). Some rumen ciliates have endosymbiotic methanogens FEMS Microbiol Lett 117 157–161 10.1111/j.1574-6968.1994.tb06758.x . 8181718

[mgen000034-] Godoy-Vitorino F. Goldfarb K. C. Brodie E. L. Garcia-Amado M. A. Michelangeli F. Domínguez-Bello M. G. (2010). Developmental microbial ecology of the crop of the folivorous hoatzin ISME J 4 611–620 10.1038/ismej.2009.147 . 20130656

[mgen000034-Good1] Good I. J. (1953). On population frequencies of species and the estimation of population parameters Biometrika 40 237–264 10.1093/biomet/40.3-4.237.

[mgen000034-Hamady1] Hamady M. Lozupone C. Knight R. (2010). Fast UniFrac: facilitating high-throughput phylogenetic analyses of microbial communities including analysis of pyrosequencing and PhyloChip data ISME J 4 17–27 10.1038/ismej.2009.97 . 19710709 PMC2797552

[mgen000034-Hristov1] Hristov A. N. (2012). Historic, pre-European settlement, and present-day contribution of wild ruminants to enteric methane emissions in the United States J Anim Sci 90 1371–1375 10.2527/jas.2011-4539 . 22178852

[mgen000034-Hundertmark1] Hundertmark K. J. Bowyer R. T. Shields G. F. Schwartz C. C. (2003). Mitochondrial phylogeography of moose (*Alces alces*) in North America J Mammal 84 718–728 10.1644/1545-1542(2003)084<0718:MPOMAA>2.0.CO;2.

[mgen000034-Hungate1] Hungate R. E. (1942). The culture of *Eudiplodinium neglectum* with experiments on the digestion of cellulose Biol Bull 83 303–319 10.2307/1538229.

[mgen000034-Imai1] Imai S. Oku Y. Morita T. Ike K. Guirong (2004). Rumen ciliate protozoal fauna of reindeer in Inner Mongolia, China J Vet Med Sci 66 209–212 10.1292/jvms.66.209 . 15031553

[mgen000034-Ishaq1] Ishaq S. L. Wright A.-D. G. (2012). Insight into the bacterial gut microbiome of the North American moose (*Alces alces*) BMC Microbiol 12 212 10.1186/1471-2180-12-212 . 22992344 PMC3585231

[mgen000034-Ishaq12] Ishaq S. L. Wright A.-D. G. (2014a). High-throughput DNA sequencing of the ruminal bacteria from moose (*Alces alces*) in Vermont, Alaska, and Norway Microb Ecol 68 185–195 10.1007/s00248-014-0399-0 . 24595908

[mgen000034-Ishaq123] Ishaq S. L. Wright A.-D. G. (2014b). Design and validation of four new primers for next-generation sequencing to target the 18S rRNA genes of gastrointestinal ciliate protozoa Appl Environ Microbiol 80 5515–5521 10.1128/AEM.01644-14 . 24973070 PMC4136085

[mgen000034-Joblin1] Joblin K. N. Naylor G. E. Williams A. G. (1990). Effect of *Methanobrevibacter smithii* on xylanolytic activity of anaerobic ruminal fungi Appl Environ Microbiol 56 2287–2295. 16348244 10.1128/aem.56.8.2287-2295.1990 PMC184724

[mgen000034-Johnson1] Johnson K. A. Johnson D. E. (1995). Methane emissions from cattle J Anim Sci 73 2483–2492. 8567486 10.2527/1995.7382483x

[mgen000034-Krascheninnikow1] Krascheninnikow S. (1955). Observations on the morphology and division of *Eudiplodinium neglectum Dogiel* (Ciliata Ento-diniomorpha) from the stomach of a moose (*Alces americana*) J Protozool 2 124–134 10.1111/j.1550-7408.1955.tb02412.x.

[mgen000034-Lechner1] Lechner I. Barboza P. Collins W. Fritz J. Günther D. Hattendorf B. Hummel J. Südekum K.-H. Clauss M. (2010). Differential passage of fluids and different-sized particles in fistulated oxen (*Bos primigenius f. taurus*), muskoxen (*Ovibos moschatus*), reindeer (*Rangifer tarandus*) and moose (*Alces alces*): rumen particle size discrimination is independent from contents stratification Comp Biochem Physiol A Mol Integr Physiol 155 211–222 10.1016/j.cbpa.2009.10.040 . 19896552

[mgen000034-Li1] Li R. W. Connor E. E. Li C. Baldwin Vi R. L. Sparks M. E. (2012). Characterization of the rumen microbiota of pre-ruminant calves using metagenomic tools Environ Microbiol 14 129–139 10.1111/j.1462-2920.2011.02543.x . 21906219

[mgen000034-Liu1] Liu C. Zhu Z. P. Liu Y. F. Guo T. J. Dong H. M. (2012). Diversity and abundance of the rumen and fecal methanogens in Altay sheep native to Xinjiang and the influence of diversity on methane emissions Arch Microbiol 194 353–361 10.1007/s00203-011-0757-y . 22038025

[mgen000034-Mathur1] Mathur R. Kim G. Morales W. Sung J. Rooks E. Pokkunuri V. Weitsman S. Barlow G. M. Chang C. Pimentel M. (2013). Intestinal *Methanobrevibacter smithii* but not total bacteria is related to diet-induced weight gain in rats Obesity (Silver Spring) 21 748–754 10.1038/oby.2012.141 . 23712978

[mgen000034-McAllister1] McAllister T. A. Okine E. K. Mathison G. W. Cheng K.-J. (1996). Dietary, environmental and microbiological aspects of methane production in ruminants Can J Anim Sci 76 231–243 10.4141/cjas96-035.

[mgen000034-Michalowski1] Michałowski T. Muszyński P. Landa I. (1991). Factors influencing the growth of rumen ciliates *Eudiplodinium maggii**in vitro* Acta Protozool 30 115–120.

[mgen000034-Morgavi1] Morgavi D. P. Martin C. Jouany J.-P. Ranilla M. J. (2012). Rumen protozoa and methanogenesis: not a simple cause-effect relationship Br J Nutr 107 388–397 10.1017/S0007114511002935 . 21762544

[mgen000034-Naga1] Naga M. A. Abou Akkada A. R. el-Shazly K (1969). Establishment of rumen ciliate protozoa in cow and water buffalo (*Bosbubalus* L.) calves under late and early weaning systems J Dairy Sci 52 110–112 10.3168/jds.S0022-0302(69)86510-0 . 5812500

[mgen000034-Newbold1] Newbold C. J. Lassalas B. Jouany J.-P. (1995). The importance of methanogens associated with ciliate protozoa in ruminal methane production *in vitro* Lett Appl Microbiol 21 230–234 10.1111/j.1472-765X.1995.tb01048.x . 7576513

[mgen000034-Ohene-Adjei1] Ohene-Adjei S. Teather R. M. Ivan M. Forster R. J. (2007). Postinoculation protozoan establishment and association patterns of methanogenic archaea in the ovine rumen Appl Environ Microbiol 73 4609–4618 10.1128/AEM.02687-06 . 17513586 PMC1932809

[mgen000034-Piao1] Piao Hailan P. Lachman M. Malfatti S. Sczyrba A. Knierim B. Auer M. Tringe S. G. Mackie R. I. Yeoman C. J. Hess M. (2014). Temporal dynamics of fibrolytic and methanogenic rumen microorganisms during *in situ* incubation of switchgrass determined by 16S rRNA gene profiling Front Microbiol 5 307. 25101058 10.3389/fmicb.2014.00307 PMC4106096

[mgen000034-Rea1] Rea S. Bowman J. P. Popo versus ki S. Pimm C. Wright A.-D. G. (2007). *Methanobrevibacter millerae* sp. nov. and *Methanobrevibacter olleyae* sp. nov., methanogens from the ovine and bovine rumen that can utilize formate for growth Int J Syst Evol Microbiol 57 450–456 10.1099/ijs.0.63984-0 . 17329767

[mgen000034-Saengkerdsub1] Saengkerdsub S. Herrera P. Woodward C. L. Anderson R. C. Nisbet D. J. Ricke S. C. (2007). Detection of methane and quantification of methanogenic archaea in faeces from young broiler chickens using real-time PCR Lett Appl Microbiol 45 629–634 10.1111/j.1472-765X.2007.02243.x . 17922818

[mgen000034-Samuel1] Samuel B. S. Gordon J. I. (2006). A humanized gnotobiotic mouse model of host-archaeal-bacterial mutualism Proc Natl Acad Sci U S A 103 10011–10016 10.1073/pnas.0602187103 . 16782812 PMC1479766

[mgen000034-Schloss1] Schloss P. D. Westcott S. L. Ryabin T. Hall J. R. Hartmann M. Hollister E. B. Lesniewski R. A. Oakley B. B. Parks D. H. other authors (2009). Introducing mothur: open-source, platform-independent, community-supported software for describing and comparing microbial communities Appl Environ Microbiol 75 7537–7541 10.1128/AEM.01541-09 . 19801464 PMC2786419

[mgen000034-Shannon1] Shannon C. E. Weaver W. (1949). The Mathematical Theory of Communication Urbana, IL University of Illinois Press.

[mgen000034-Sharp1] Sharp R. Ziemer C. J. Stern M. D. Stahl D. A. (1998). Taxon-specific associations between protozoal and methanogen populations in the rumen and a model rumen system FEMS Microbiol Ecol 26 71–78 10.1111/j.1574-6941.1998.tb01563.x.

[mgen000034-Shipley1] Shipley L. A. Blomquist S. Danell K. (1998). Diet choices by free-ranging moose in northern Sweden in relation to plant distribution, chemistry, and morphology Can J Zool 76 1722–1733 10.1139/z98-110.

[mgen000034-Skillman1] Skillman L. C. Evans P. N. Naylor G. E. Morvan B. Jarvis G. N. Joblin K. N. (2004). 16S ribosomal DNA-directed PCR primers for ruminal methanogens and identification of methanogens colonising young lambs Anaerobe 10 277–285 10.1016/j.anaerobe.2004.05.003 . 16701528

[mgen000034-Sladecek1] Sládeček F (1946). Ophryoscolecidae from the stomach of *Cervus elaphus* L., *Dama dama* L., and *Capreolus capreolus* L Vestn Csl Zool Spole 10 201–231.

[mgen000034-Snelling1] Snelling T. J. Genç B. McKain N. Watson M. Waters S. M. Creevey C. J. Wallace R. J. (2014). Diversity and community composition of methanogenic archaea in the rumen of Scottish upland sheep assessed by different methods PLoS One 9 e106491 10.1371/journal.pone.0106491 . 25250654 PMC4175461

[mgen000034-St-Pierre1] St-Pierre B. Wright A.-D. G. (2012). Molecular analysis of methanogenic archaea in the forestomach of the alpaca (*Vicugna pacos*) BMC Microbiol 12 1 10.1186/1471-2180-12-1 . 22221383 PMC3292460

[mgen000034-Stumm1] Stumm C. K. Gijzen H. J. Vogels G. D. (1982). Association of methanogenic bacteria with ovine rumen ciliates Br J Nutr 47 95–99 10.1079/BJN19820013 . 6800402

[mgen000034-Sundset1] Sundset M. A. Praesteng K. E. Cann I. K. O. Mathiesen S. D. Mackie R. I. (2007). Novel rumen bacterial diversity in two geographically separated sub-species of reindeer Microb Ecol 54 424–438 10.1007/s00248-007-9254-x . 17473904

[mgen000034-Sundset12] Sundset M. A. Edwards J. E. Cheng Y. F. Senosiain R. S. Fraile M. N. Northwood K. S. Praesteng K. E. Glad T. Mathiesen S. D. Wright A.-D. G. (2009a). Rumen microbial diversity in Svalbard reindeer, with particular emphasis on methanogenic archaea FEMS Microbiol Ecol 70 553–562 10.1111/j.1574-6941.2009.00750.x . 19702875

[mgen000034-Sundset123] Sundset M. A. Edwards J. E. Cheng Y. F. Senosiain R. S. Fraile M. N. Northwood K. S. Praesteng K. E. Glad T. Mathiesen S. D. Wright A.-D. G. (2009b). Molecular diversity of the rumen microbiome of Norwegian reindeer on natural summer pasture Microb Ecol 57 335–348 10.1007/s00248-008-9414-7 . 18604648

[mgen000034-Sylvester1] Sylvester J. T. Karnati S. K. R. Yu Z. Morrison M. Firkins J. L. (2004). Development of an assay to quantify rumen ciliate protozoal biomass in cows using real-time PCR J Nutr 134 3378–3384. 15570040 10.1093/jn/134.12.3378

[mgen000034-Vogels1] Vogels G. D. Hoppe W. F. Stumm C. K. (1980). Association of methanogenic bacteria with rumen ciliates Appl Environ Microbiol 40 608–612. 6775596 10.1128/aem.40.3.608-612.1980 PMC291627

[mgen000034-Wam1] Wam H. K. Hjeljord O. (2010). Moose summer and winter diets along a large scale gradient of forage availability in southern Norway Eur J Wildl Res 56 745–755 10.1007/s10344-010-0370-4.

[mgen000034-Westerling1] Westerling B. (1969). The rumen ciliate fauna of cattle and sheep in Finnish Lapland, with special reference to the species regarded as specific to reindeer Nord Vet Med 21 14–19.

[mgen000034-Williams1] Williams A. G. Coleman G. S. (1992). Metabolism of Entodiniomorphid Protozoa in the Rumen Protozoa New York Springer.

[mgen000034-Wright1] Wright A.-D. G. Klieve A. V. (2011). Does the complexity of the rumen microbial ecology preclude methane mitigation? Anim Feed Sci Technol 166-167 248–253 10.1016/j.anifeedsci.2011.04.015.

[mgen000034-Wright12] Wright A.-D. G. Williams A. J. Winder B. Christophersen C. T. Rodgers S. L. Smith K. D. (2004). Molecular diversity of rumen methanogens from sheep in Western Australia Appl Environ Microbiol 70 1263–1270 10.1128/AEM.70.3.1263-1270.2004 . 15006742 PMC368393

[mgen000034-Wright123] Wright A.-D. G. Ma X. Obispo N. E. (2008). *Methanobrevibacter* phylotypes are the dominant methanogens in sheep from Venezuela Microb Ecol 56 390–394 10.1007/s00248-007-9351-x . 18165875

[mgen000034-Wright1234] Wright A.-D. G. Northwood K. S. Obispo N. E. (2009). Rumen-like methanogens identified from the crop of the folivorous South American bird, the hoatzin (*Opisthocomus hoazin*) ISME J 3 1120–1126 10.1038/ismej.2009.41 . 19387486

[mgen000034-Zhou1] Zhou M. Hernandez-Sanabria E. Guan L. L. (2010). Characterization of variation in rumen methanogenic communities under different dietary and host feed efficiency conditions, as determined by PCR-denaturing gradient gel electrophoresis analysis Appl Environ Microbiol 76 3776–3786 10.1128/AEM.00010-10 . 20418436 PMC2893468

